# Targeted hepatitis C antibody testing interventions: a systematic review and meta-analysis

**DOI:** 10.1007/s10654-014-9958-4

**Published:** 2014-11-11

**Authors:** Esther Jane Aspinall, Joseph Samuel Doyle, Stephen Corson, Margaret Elena Hellard, David Hunt, David Goldberg, Tim Nguyen, Yngve Falck-Ytter, Rebecca Lynn Morgan, Bryce Smith, Mark Stoove, Stefan Zbyszko Wiktor, Sharon Hutchinson

**Affiliations:** 1 School of Health and Life Sciences, Glasgow Caledonian University, Glasgow, G4 0BA UK; 2 Health Protection Scotland, 5 Cadogan Street, Glasgow, G2 6QE UK; 3 Burnet Institute, 85 Commercial Road, Melbourne, 3004 Australia; 4 School of Public Health and Preventive Medicine, Monash University, Melbourne, 3800 Australia; 5 Department of Infectious Diseases, Alfred Health, Melbourne, 3181 Australia; 6 Department of Mathematics and Statistics, University of Strathclyde, Glasgow, G1 1XQ UK; 7 Global Hepatitis Programme, World Health Organization, 1211 Geneva 27, Switzerland; 8 Case and VA Medical Center, Case Western Reserve University, Cleveland, OH 44106 USA; 9 Centers for Disease Control and Prevention, Atlanta, GA 30333 USA

**Keywords:** Hepatitis C, Testing, Systematic review, Meta-analysis

## Abstract

Testing for hepatitis C virus (HCV) infection may reduce the risk of liver-related morbidity, by facilitating earlier access to treatment and care. This review investigated the effectiveness of targeted testing interventions on HCV case detection, treatment uptake, and prevention of liver-related morbidity. A literature search identified studies published up to 2013 that compared a targeted HCV testing intervention (targeting individuals or groups at increased risk of HCV) with no targeted intervention, and results were synthesised using meta-analysis. Exposure to a targeted testing intervention, compared to no targeted intervention, was associated with increased cases detected [number of studies (n) = 14; pooled relative risk (RR) 1.7, 95 % CI 1.3, 2.2] and patients commencing therapy (n = 4; RR 3.3, 95 % CI 1.1, 10.0). Practitioner-based interventions increased test uptake and cases detected (n = 12; RR 3.5, 95 % CI 2.5, 4.8; and n = 10; RR 2.2, 95 % CI 1.4, 3.5, respectively), whereas media/information-based interventions were less effective (n = 4; RR 1.5, 95 % CI 0.7, 3.0; and n = 4; RR 1.3, 95 % CI 1.0, 1.6, respectively). This meta-analysis provides for the first time a quantitative assessment of targeted HCV testing interventions, demonstrating that these strategies were effective in diagnosing cases and increasing treatment uptake. Strategies involving practitioner-based interventions yielded the most favourable outcomes. It is recommended that testing should be targeted at and offered to individuals who are part of a population with high HCV prevalence, or who have a history of HCV risk behaviour.

**Electronic supplementary material:**

The online version of this article (doi:10.1007/s10654-014-9958-4) contains supplementary material, which is available to authorized users.

## Introduction

It is estimated that 185 million people have been infected with hepatitis C virus (HCV) worldwide [1], most of whom are unaware of their infection [2]. The burden is highest in low- and middle-income countries (LMIC), which account for over 80 % of cases of chronic HCV infection [1]. People living with HCV may experience considerable barriers to accessing testing, treatment and care, particularly in low-income countries [3, 4].

Populations at increased risk of HCV include people who inject drugs (PWID) [5], people receiving medical procedures (including transfusion of blood and blood products) in an unsafe setting [6, 7], men who have sex with men (MSM) (in particular, those who are infected with HIV) [8], and children born to mothers who have HCV [9]. Intranasal drug use and cosmetic procedures (such as tattooing, body piercing, and manicures) have also been implicated as risk factors for HCV [10]. The relative importance of these risk factors varies depending on the geographical setting and population studied.

Chronic HCV infection leads to an increased risk of liver cirrhosis and liver cancer, and contributes to approximately 360,000 liver-related deaths annually [11, 12]. Testing for and diagnosis of HCV is expected to reduce the risk of liver-related disease, by facilitating earlier access to HCV treatment and care. On this basis, European and American HCV guidelines have recommended targeted HCV testing for high risk groups, without necessarily the evidence to demonstrate that early diagnosis is of benefit [13–15].

A recent narrative synthesis of eight studies concluded that testing interventions can lead to increases in test uptake, but other outcomes were not examined in detail [16]. The aim of our review was to investigate and quantify through meta-analysis the effectiveness of targeted HCV testing interventions on patient-important outcomes, including test uptake, case detection, uptake of HCV treatment, and the prevention of liver-related morbidity and mortality. The study was conducted as part of a series of systematic reviews to inform World Health Organisation (WHO) Guidelines on the Screening, Care, and Treatment of Hepatitis C, with particular reference to LMIC [17].

## Materials and methods

### Literature search and data extraction

**Fig. 1:**
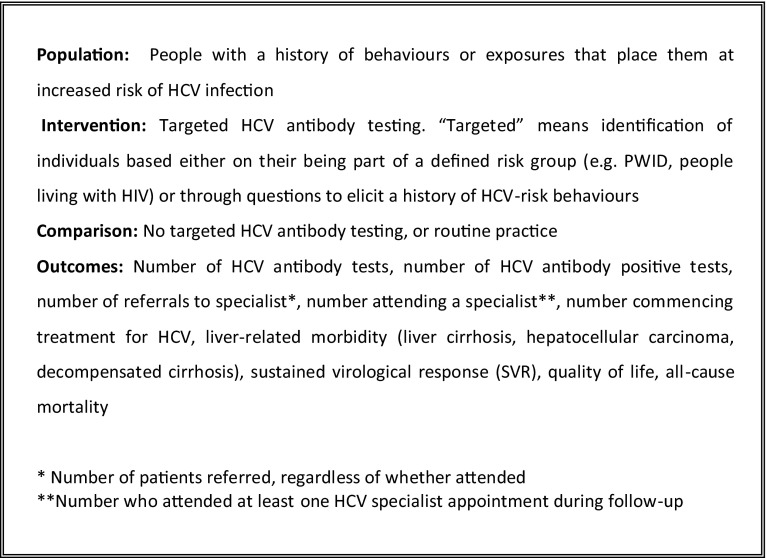
Population, intervention, comparison, and outcome (PICO) inclusion criteria

The review was prospectively registered with PROSPERO (registration number CRD42013004146). A literature search was undertaken to identify relevant articles in any language published between January 1994 and March 2013 in the following databases: Medline, Embase, LILACS, Cochrane library of Systematic Reviews, the NHS Economic Evaluations Database (NHS EDD), Health Technology Assessments Database (HTA), Database of Abstracts of Reviews of Effects (DARE), and the European Network of Health Economic Evaluations Database (EURONHEED). Search syntax is shown in Appendix 1, briefly summarized as: Hepatitis C AND test, case-finding, or screening. Reference lists of relevant articles were checked for additional papers. Relevant cost effectiveness studies were reviewed to check for empirical data that met the inclusion criteria (Fig. 1). Foreign language articles were translated online using Google Translate (Google, Palo Alto USA, 2013). Due to the large number of citations (>10,000), a single reviewer conducted the citation screening, and two reviewers carried out abstract and full-text screening. A third reviewer was consulted on any points of difference between the first and second reviewer.

Studies were included if they compared a targeted testing intervention with no targeted intervention, or routine practice (Fig. 1). Included studies were assessed for quality using the Cochrane Risk of Bias tool [18]. Missing data on outcomes of interest were requested from primary authors, with each author contacted twice in the case of non-response. Data were extracted on study design, setting, year, population, sample size, selection and characteristics of the intervention and comparison group, type of intervention, and any of the following outcomes (for both the intervention and comparison groups): number tested for HCV antibody, detected as HCV antibody positive, referred to and attended specialist care, initiated on HCV treatment, attained a SVR, developed cirrhosis/hepatocellular carcinoma (HCC), and died from a liver-related cause.

### Population denominators

The population denominator for the two testing outcomes (test uptake and HCV antibody cases detected) was the number of people eligible to receive the testing intervention. The definition of the ‘eligible population’ varied depending on the type of study: in the practitioner-based studies, it was possible to record the number of people eligible for or offered the intervention. However, for the media/information-based studies, the number of people exposed to the intervention was not known, and the eligible population was therefore defined as the number of people residing in the region where the intervention took place. If this was not provided, the eligible population size was estimated from other information provided in the study (e.g. the number of GP practices in the area), and sensitivity analyses were conducted around the lowest likely and highest likely population size.

For the treatment and care outcomes (referral/attendance at specialist, treatment uptake, and SVR), where HCV positivity was a pre-requisite for achieving that outcome, the denominator was the estimated number of people in the eligible population who were HCV antibody positive. This was calculated in two stages: (1) first, HCV prevalence in the study population was estimated, and (2) this prevalence was then applied to the eligible population in the intervention and comparison groups. In stage (1), HCV prevalence was estimated as the mid-point between the lowest possible prevalence (calculated as: number of HCV antibody positive cases detected/eligible population) and the highest likely prevalence, assuming that the tested population was more likely to be HCV positive than the untested population (calculated as: number of HCV antibody positive cases detected/number tested), both among the intervention group. The rationale for using the intervention group to estimate HCV prevalence was that HCV testing in the comparison group was more likely to relate to individuals presenting with symptomatic disease, and thus could over-estimate the prevalence of HCV in the eligible population. In addition, some studies did not report the number of HCV antibody positive cases in the comparison group.

### Data synthesis

Pooled relative risks (RR) were calculated using random effects meta-analysis (Inverse Variance [IV] method). Heterogeneity was assessed using both I^2^ and stratified analyses of the following subgroups:

1. ‘Practitioner-based targeted testing’ (defined as interventions where a health or social care practitioner was given in-practice support to offer risk assessment and/or HCV testing) versus ‘Media/information-based targeted testing’ (defined as interventions comprising of television, radio or newspaper advertisements, provision of posters or leaflets, or invitations to information sessions for practitioners or people at risk).
2. Testing targeted at individuals known to be PWID (e.g. identifying PWID through medical records, or offering tests at services for PWID), versus testing targeted at groups at increased risk of being PWID (e.g. specific birth cohorts, homeless populations, prisoners, psychiatric inpatients), or testing targeted more broadly at all risk groups.

Sensitivity analyses were conducted to assess the effect on the pooled effect estimates of:

1. Type of study design [randomised controlled trials (RCT) vs. non-RCT]
2. Inclusion/exclusion of individual studies of interest
3. Estimating the eligible population denominator for studies where this was not provided: (best estimate, versus the lowest likely denominator or the highest likely denominator).
4. Estimating HCV prevalence for the studies that reported on treatment and care outcomes: (best estimate, versus the lowest possible prevalence or the highest likely prevalence).

Anticipated absolute effects of the intervention (i.e. the number of additional cases detected, referred, attended specialist, commenced therapy, and achieved a SVR) were calculated for:

1. The pooled study population
2. Hypothetical populations with HCV prevalence of either 10 or 50 %.

All statistical analyses were carried out using Review Manager Version 5.2 (Cochrane Collaboration, Copenhagen) and GRADE Profiler Version 3.6 (GRADE Working Group).

### Outcome assessments

The quality of evidence for each outcome was assessed using GRADE (Grading of Recommendations Assessment, Development and Evaluation) [19].

## Results

### Characteristics of the included studies

**Fig. 2:**
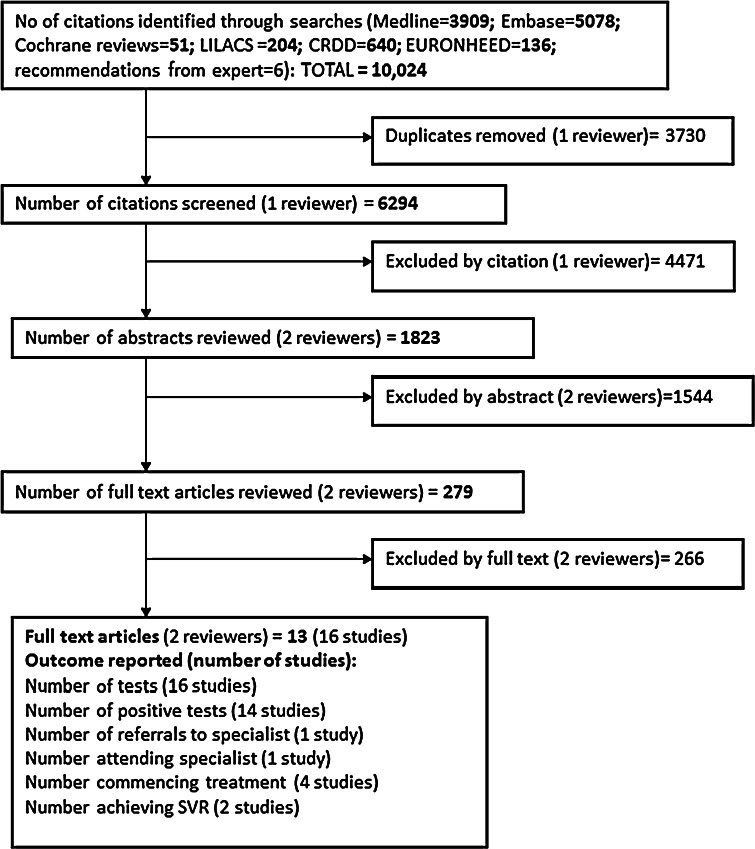
Flowchart of study selection for the systematic review

Results of the literature search are shown in Fig. 2. Thirteen articles and one conference abstract met the inclusion criteria, but one article was excluded [20] because a more recent article on the same study population was identified [21]. Three articles [22–24] reported two distinct studies within the same article; therefore there were sixteen studies in total.

The sixteen included studies are shown in Table 1. There were five cluster RCTs [24–27], all of which were assessed as having low risk of bias. There were eleven non-RCTs, all of which were assessed as having high risk of bias. Four were controlled trials [28–31], three were before/after studies [23, 32], and four were time-series analyses [21, 22, 33].

**Table 1:** Characteristics of sixteen studies included in the systematic review

Primary author, year, location	Setting	Study design, (follow-up)	Target population	Eligible population	Intervention	Comparison
Anderson [28] UK	Two general practices in area of socio-economic deprivation	Non-randomised controlled trial (4 years)	Birth cohort living in area of socioeconomic deprivation	Patients aged 30–54 years attending non-urgent GP appointments	Patients were offered a test and given an information leaflet. Those accepting the offer could attend testing and counselling immediately, or return at a later date	People attending a comparison practice received routine care
Cullen [25] Ireland	25 General practices where at least one GP prescribed methadone	Cluster randomised controlled trial (6 months)	Current/former PWID	Patients receiving methadone from their GP	A liaison support nurse discussed screening guidelines with practice staff, provided clinical and administrative support, liaised with hepatology and addiction services, and carried out testing at practices	Control practices continued with routine care
Cullen [29] UK	16 General practices serving area of socioeconomic deprivation	Non-randomised controlled trial (3 years)	Birth cohort of current/former PWID	Patients aged 30–54 years (with records suggesting PWID) at non-urgent appointments	Patients were offered a test and given an information leaflet. Participants returned to the practice to receive results and post-test discussion from their GP. One General Practice received a staff seminar, and the remaining seven received HCV information	Control practices continued with routine practice, and were not aware of their participation in the trial
Defossez [21] France	Poitou–Charentes region, population 1.6 million	Time series analysis (6 years)	All people at increased risk	Population residing in intervention area	National programme commenced in June 1999, which included implementation of a targeted screening programme and repeated media campaigns	The same population prior to roll-out of the intervention
Helsper [30] The Netherlands	219 General practices across two regions of the Netherlands	Non-randomised controlled trial (N/R)	All people at increased risk	Population residing in the intervention area	A support campaign for GPs, which included education sessions and in-practice support from practice facilitators to carry out HCV risk assessment. A concurrent public campaign (radio/newspaper ads, information distribution) was implemented in both intervention and control regions	Control practices continued with routine care. Control region was exposed to the same public (media) campaign as the intervention region
Helsper [22] (a) The Netherlands	Gelre-UJssel region, population 166,315	Time series analysis (4 months)	All people at increased risk	Population residing in the intervention area	Radio and newspaper advertisements, distribution of specially designed posters and brochures in public areas where risk groups were expected to congregate	The same population prior to roll-out of the intervention
Helsper [22] (b), The Netherlands	Drug services in Rotterdam	Time series analysis (5 months)	‘Hard drug users’ (HDU)	Estimated population of HDU living in Rotterdam	26 addictions professionals were trained to provide HCV counselling, which was actively offered to HDU. Three information meetings were attended by 180 HDU	The same population prior to roll-out of the intervention
Hickman [26] Multi-site, UK	14 specialist drug clinics and six prisons	Cluster randomised controlled trial (N/R)	Current PWID and prisoners	Drug users at specialist drug clinics, or prison inmates	HCV testing using dried blood spot (DBS) test. Staff training and information on DBS, plus on-going support from local specialist HCV nurses	Matched prison or drug services received routine care
Lacey [32] Australia	Inpatient psychiatry unit at tertiary hospital	Before/after study (N/R)	Psychiatric in-patients	All patients admitted to psychiatric unit	A leaflet providing information on HCV was distributed, and a research assistant facilitated education/discussion groups, and carried out counselling and testing	Patients admitted to the same unit prior to the intervention
Lewis [31] UK	GP practices serving Pakistani population	Non-randomised controlled trial (N/R)	South Asian migrant population in UK	South Asian patients registered with GP practices	Patients were invited by letter to opt-out of screening. Those who did not opt-out were asked to attend screening clinics held by Hepatologyteam at GP surgeries	South Asian patients were offered HCV testing if they attended the GP practice
Litwin [23] (a) USA	Three primary care clinics in area of socio-economic deprivation	Before/after study (N/R)	All people at increased risk living in an area of deprivation	Patients attending primary care clinics	Researchers placed a ‘risk sticker’ on patient case notes, which prompted medical staff to ask about HCV risk factors, and to offer testing if any risk factors	Patients attending the same practices prior to the intervention
Litwin [23] (b) USA	Threeprimary care clinics in area of socio-economic deprivation	Before/after study (N/R)	Birth cohort living in an area of socio-economic deprivation	Patients born between 1945 and 1964 and attending primary care clinics	Researchers placed a ‘birth cohort sticker’ on patient case notes, which prompted medical staff to offer HCV testing to all patients born between 1945 and 1964	Patients attending the same practices prior to the intervention
Roudot-Thoraval [27] France	184 General practices in the Creteil region	Cluster randomised controlled trial (N/R)	All people at increased risk	Population residing in the intervention area	Provision of posters and leaflets in GP surgeries, informing patients of the risk factors for HCV	Patients attending GP surgeries where the posters and leaflets were not provided
Sahajian [33] France	3,052 General practitioners and private practices in Lyon region	Time series analysis (12 months)	All people at increased risk	Population residing in the intervention area	A guide on HCV testing was mailed to private practitioners. GPs and laboratory physicians were invited to workshops and training sessions on HCV testing	Population of the same region prior to the roll-out of the intervention
Sahajian [24] (a) France	12 Homeless hostels providing long-term accommodation	Cluster randomised controlled trial (N/R)	Homeless population	Individuals staying at homeless hostels	Group information sessions for residents were followed by referral, if interested, to a Health Centre where a medical check-up and HCV testing were carried out	Individuals staying at comparison shelters received routine care
Sahajian [24] (b) France	12 Homeless hostels providing long-term accommodation	Cluster randomised controlled trial (N/R)	Homeless population	Individuals staying at homeless hostels	Group information sessions were followed by on-site medical check-ups and HCV testing for those who were interested	Individuals staying at comparison shelters received routine care

*N/R* not reported

Twelve studies involved practitioner-based targeted interventions [22–26, 28–32] while the remaining four involved media/information-based targeted interventions [21, 22, 27, 33]. Four studies targeted individuals with a history of PWID, either through use of drug services [22, 25, 26], or review of medical records [29]. Five studies targeted groups at increased risk of being PWID, which included homeless populations [24], psychiatric inpatients [32], and individuals within a specified birth cohort and residing in an area of socio-economic deprivation [23, 28]. Six studies targeted people with any risk factor for HCV, either by prompting practitioners to question their patients on a list of risk factors for HCV [23, 30], or through media campaigns advising people at risk to present for testing [21, 22, 27, 33]. The remaining study targeted a South Asian community living in the UK [31].

### Findings of the studies

Sixteen studies reported on test uptake and fourteen reported on HCV antibody positive cases detected in both the intervention and comparison groups (Table 2). Most studies reported that testing interventions increased the number of tests and the number of cases detected, except Lacey et al. and Helsper et al. [22] (b) (which did not report on case detection in the comparison group) and Roudot-Thoraval et al. (where uptake and case detection decreased); the latter study provided information leaflets and posters about HCV risk factors to randomly selected GP surgeries, and continued with routine practice in the comparison practices. The number of individuals needed to test to detect a single HCV antibody positive case varied depending on the population group targeted for testing: it was highest when all risk groups were targeted (range 19–118), and lowest when either groups at increased risk of being PWID (8–36) or individual PWID (range 1–4) were targeted.

**Table 2:** Outcomes of HCV testing interventions in sixteen studies included in the systematic review

Primary author, year	Study group	Time period	Number in eligible population	Number tested	Number HCV antibody positive	Number tested to detect one case	Number referred	Number attended	Number started treatment	Number achieved SVR	Estimated HCV prevalence (range)^b^
Anderson [28]	Intervention	2003–2004	584	117	15	8	11	11	2	1	7.7 % (2.6–12.8 %)
	Comparison	2003–2004	458	0	0	No cases	0	0	0	0	
Cullen [25]	Intervention	NA	104	51	73^a^	NA	44	37	5	–	NA (70.2 % to NA)
	Comparison	NA	92	25	41^a^	NA	13	9	1	–	
Cullen [29]	Intervention	2007	485	105	74	1	31	22	4	2	42.9 % (15.3–70.5 %)
	Comparison	2007	528	36	8	5	3	2	2	2	
Defossez [21]	Intervention	2003	1,677,855	20,920	307	68	–	–	–	–	0.7 % (0.0–1.5 %)
	Comparison	1997	1,640,453	6,168	196	31	–	–	–	–	
Helsper [30]	Intervention	2007–2008	269,125	172	3	57	–	–	–	–	0.9 % (0.0–1.7 %)
	Comparison	2007–2008	266,678	118	1	118	–	–	–	–	
Helsper [22] (a)	Intervention	2007–2008	166,315	118	1	118	–	–	–	–	0.4 % (0.0–0.8 %)
	Comparison	2007	166,315	86	0	No cases	–	–	–	–	
Helsper [22] (b)	Intervention	2007–2008	5,000	186	57	3	–	–	–	–	15.9 % (1.1–30.6 %)
	Comparison	2007	5,000	~ 0	NA	NA	–	–	–	–	
Hickman [26]	Intervention	2004–2005	6,550	791	216	4	–	–	–	–	15.3 % (3.3–27.3 %)^c^
	Comparison	2004–2005	5,800	243	104	2	–	–	–	–	
Lacey [32]	Intervention	2002–2003	402	71	14	5	–	–	–	–	11.6 % (3.5–19.7 %)
	Comparison	2002	430	40	NA	NA	–	–	–	–	
Lewis [31]	Intervention	NA	1,163	229	5	45	5	5	2	–	1.3 % (0.4–2.2 %)
	Comparison	NA	1,134	17	0	No cases	0	0	0	–	
Litwin [23] (a)	Intervention	2008–2009	8,981	1,179	62	19	–	–	–	–	3.0 % (0.7–5.3 %)
	Comparison	2008	6,591	394	36	11	–	–	–	–	
Litwin [23] (b)	Intervention	2009	10,165	1,008	59	17	–	–	–	–	3.2 % (0.6–5.9 %)
	Comparison	2008	6,591	394	36	11	–	–	–	–	
Roudot-Thorval [27]	Intervention	1997–1998	~94,000	294	10	29	–	–	–	–	1.7 % (0.0–3.4 %)
	Comparison	1997–1998	~90,000	323	15	22	–	–	–	–	
Sahajian [33]	Intervention	2000–2001	1.5 m	15,952	276	58	–	–	–	–	0.9 % (0.0–1.7 %)
	Comparison	1999–2000	1.5 m	13,799	231	60	–	–	–	–	
Sahajian [24] (a)	Intervention	2007–2009	222	95	3	32	–	–	–	–	2.3 % (1.4–3.2 %)
	Comparison	2007–2009	811	12	0	No cases	–	–	–	–	
Sahajian [24] (b)	Intervention	2007–2009	784	145	4	36	–	–	–	–	1.6 % (0.5–2.8 %)
	Comparison	2007–2009	811	12	0	No cases	–	–	–	–	

*NA* not available

^a^Patients testing HCV positive during the time period of the study but in non-study settings were included; therefore the number of positive tests exceeds the total number tested

^b^Estimated as the mid-point between the lowest possible prevalence (number HCV antibody positive/number eligible) and the highest likely prevalence (number HCV antibody positive/number tested) in the intervention group

^c^Hickman et al. targeted both drug users and prisoners: estimated HCV prevalence among drug users was 16.8 %, and among prisoners was 13.7 %

Four studies reported on treatment and care outcomes, all of which involved practitioner-based interventions [25, 28, 29, 31]. All reported an increase in the number of referrals, attendances, and treatment uptake in the intervention compared to the comparison group. Across the four intervention groups, 167 individuals were diagnosed as HCV antibody positive (including both those RNA positive and negative), of which 91 were referred to a specialist, 75 attended, and 13 commenced HCV treatment within a median of 2 years of follow-up. Assuming that 70 % of HCV antibody positive individuals were HCV RNA positive [34] (as HCV RNA results were not available for all studies), the aforementioned results would equate to 78, 64, and 11 % of patients with chronic HCV being referred, attending, and commencing treatment, respectively.

### Relative effects of targeted testing interventions

Exposure to a targeted testing intervention, compared to no targeted intervention, was associated with increased number of people tested for HCV [number of studies (n) = 16, pooled RR 2.9, 95 % confidence interval (CI) 2.0, 4.2; I^2^ = 100 %], HCV antibody cases detected (n = 14; pooled RR 1.7, 95 % CI 1.3, 2.2; I^2^ = 76 %), referrals to a specialist (n = 1; RR 3.0, 95 % CI 1.8, 5.1), attendance with a specialist (n = 1; RR 3.7, 95 % 1.9, 7.0), and cases commencing treatment (n = 4; RR 3.3, 95 % CI 1.1, 10.0; I^2^ = 0 %) (Table 3). Of the studies which reported on the number of patients achieving a SVR (over an average of 2 years of follow-up), there was no significant difference (n = 2; RR 1.4, 95 % CI 0.3, 7.1; I^2^ = 0 %) in targeted, compared to no targeted HCV testing intervention. The synthesised evidence for both the test uptake and cases detected outcomes was rated as moderate quality, because the evidence was derived from RCTs and observational studies (with minimal impact of study design on effect size—see Appendix 2), but study effect sizes were inconsistent. The synthesised evidence for the referral, attendance, and treatment outcomes was also rated as moderate quality, because the evidence was derived mainly from RCTs, but the available data was sparse.

**Table 3:** Pooled relative and absolute effects of HCV testing interventions

Outcome (median length of follow-up)	Population (studies)	Effect size (95 % CI)	I^2^	Baseline risk per 10,000 population	Anticipated absolute effects per 10,000 population (95 % CI)	Anticipated absolute effects per 10,000 if HCV prevalence is 10 %	Anticipated absolute effects per 10,000 if HCV prevalence is 50 %
Tested for HCV among the eligible population (N/A)	7,435,283 (16 studies)	**2.90 (2.01, 4.17)**	100 %	59 tests conducted^a^	112 more HCV antibody tests (from 59 more to 186 more)^a^	N/A	N/A
HCV positive cases detected among the eligible population (N/A)	7,424,451 (14 studies)	**1.66 (1.27, 2.16)**	76 %	2 cases detected^a^	1 more case detected (from 0 more to 2 more)^a^	5 more cases detected (from 2 more to 8 more)	23 more cases detected(from 9 more to 40 more)
Referral to specialist among HCV positive population (6 months)	138 (1 study)	**3.01 (1.79, 5.07)**	N/A	2,000 referrals to specialist^b^	4,020 more referrals (from 1,580 more to 8,140 more)^b^	433 more referrals (from 157 to 913 more)	1,298 more referrals (from 470 to 2,739 more)
Attendance at specialist among HCV positive population (6 months)	138 (1 study)	**3.66 (1.92, 6.99)**	N/A	1,385 attending a specialist^b^	3,683 more attendances (from 1,274 more to 8,294 more)^b^	287 more attendances (from 94 to 665 more)	1,722 more attendances(from 561 to 3,991 more)
Commenced treatment among HCV positive population (2 years)	683 (4 studies)	**3.25 (1.06, 9.95)**	0 %	88 commencing treatment^b^	197 more commencing (from 53 more to 785 more)^b^	17 more commencing (from 0 more to 67more)	67 more commencing (from 1 more to 268 more)
SVR among HCV positive population (3 years, 6 months)	515 (2 studies)	1.35 (0.26, 7.09)	0 %	76 achieving an SVR^b^	27 more achieving SVR (from 56 fewer to 465 more)^b^	2 more SVRs (from 5 fewer to 43 more)	9 more SVRs (from 21 fewer to 170 more)

Bold type denotes *p* value < 0.05

*N/A* not applicable

^a^Per 10,000 population eligible for testing

^b^Per 10,000 HCV positive population

### Sensitivity analyses (Appendix 2)

1. Inclusion of non-RCT evidence potentially over-estimated the effect estimate for two outcomes—referral to specialist, and attendance with a specialist—and so data synthesis for these outcomes was thereafter restricted to RCT evidence.
2. Inclusion of Defossez [21], which used a different length of follow-up for the pre- and post-intervention periods, had minimal impact on pooled relative risks and heterogeneity, and therefore was included in data synthesis.
3. Varying the size of the eligible population denominator across a range of likely values (for Roudot-Thoraval [27], where the size of the eligible population was not known) had no impact on pooled effect size or heterogeneity, and therefore the best estimate of the denominator was used.
4. Estimating HCV prevalence across a likely range of values (for the studies reporting on HCV treatment and care outcomes) had minimal impact on pooled effect sizes and heterogeneity, and therefore the best estimate of HCV prevalence was used.

### Stratified analyses

**Fig. 3:**
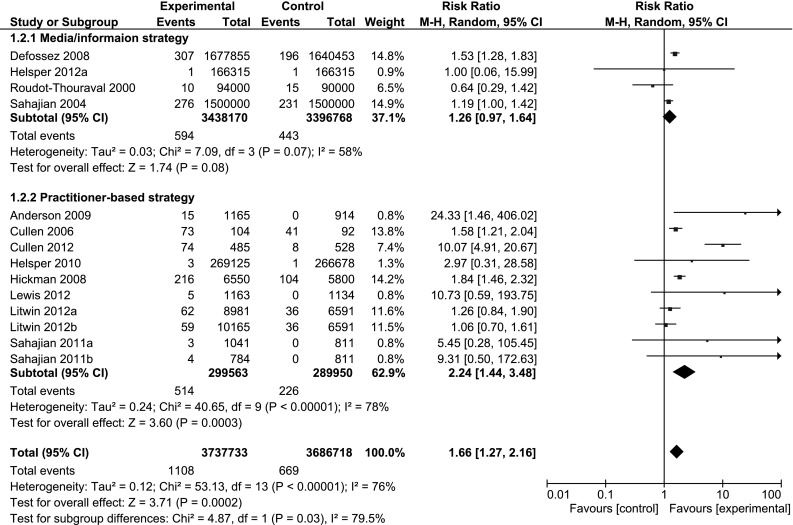
Forest plots comparing targeted HCV testing interventions versus no targeted testing intervention by type of targeted testing: outcome; HCV antibody cases detected

A practitioner-based approach to targeted testing, compared to no targeted testing, increased both the number of people tested for HCV and the number who tested positive for HCV (n = 12; RR 3.5, 95 % CI 2.5, 4.8; I^2^ = 94 %, and n = 10; 2.2, 95 % CI 1.4, 3.5; I^2^ = 78 % respectively) (Table 4). A media/information-based approach to targeted testing, compared to no targeted testing, was less effective in increasing the number of people tested for HCV and the number who tested positive (n = 4; RR 1.5, 95 % CI 0.7, 3.0; I^2^ = 100 %, and n = 4; 1.3, 95 % CI 1.0, 1.6; I^2^ = 58 % respectively) (Fig. 3).

**Table 4:** Stratified analysis

Outcome	Stratification	Subgroup	No. of studies	Studies included	Effect size (95 % CI)	Heterogeneity (I^2^) (%)
Tested for HCV	Type of targeted testing	Practitioner-based	12	Anderson [28], Cullen [25], Cullen [29], Helsper [30], Helsper [22] (b), Hickman [26], Lacey [32], Lewis [31], Litwin [23] (a, b), Sahajian [24] (a, b)	**3.47 (2.52, 4.79)**	94
		Media/information-based	4	Defossez [21], Helsper [22] (a), Roudot-Thouraval [27], Sahajian [33]	1.47 (0.71, 3.03)	100
	Target group	Individuals known to be PWID^a^	4	Cullen [25], Cullen [29], Helsper [22] (b), Hickman [26]^c^	**3.43 (1.73, 6.80)**	91
		Groups at increased risk of being PWID^b^	6	Anderson [28], Hickman [26]^c^, Lacey [32], Litwin [23] (b), Sahajian [24] (a, b)	**5.61 (2.75, 11.44)**	97
		All HCV risk groups	6	Defossez [21], Helsper [30], Helsper [22] (a), Litwin [23] (a), Roudot-Thoraval [27], Sahajian [33]	1.57 (0.89, 2.77)	100
HCV positive cases detected	Type of targeted testing	Practitioner-based	10	Anderson [28], Cullen [25], Cullen [29], Helsper [30], Hickman [26], Lewis [31], Litwin [23] (a, b), Sahajian [24] (a, b)	**2.24 (1.44, 3.48)**	78
		Media/information-based	4	Defossez [21], Helsper [22] (a), Roudot-Thouraval [27], Sahajian [33]	1.26 (0.97, 1.64)	58
	Target group	Individuals known to be PWID^a^	3	Cullen [25], Cullen [29], Helsper [22] (b), Hickman [26]^c^	**3.12 (1.37, 7.11)**	93
		Groups at increased risk of being PWID^b^	5	Anderson [28], Hickman [26]^c^, Litwin [23] (b), Sahajian [24] (a, b)	1.81 (0.91, 3.59)	65
		All HCV risk groups	6	Defossez [21], Helsper [30], Helsper [22] (a), Litwin [23] (a), Roudot-Thoraval [27], Sahajian [33]	**1.30 (1.07, 1.57)**	36

Stratification for referral, attendance, treatment commencement and SVR outcomes was not attempted due to the small number of studies

Bold type denotes *p* value < 0.05

^a^Identified through services for PWID or by review of medical records

^b^Includes the following groups: homeless, prisoners, psychiatric inpatients, birth cohort living in an area of socio-economic deprivation

^c^Hickman [26] studied two different groups (PWID at drug services, and prisoners) and therefore results are stratified for this subgroup analysis

**Fig. 4:**
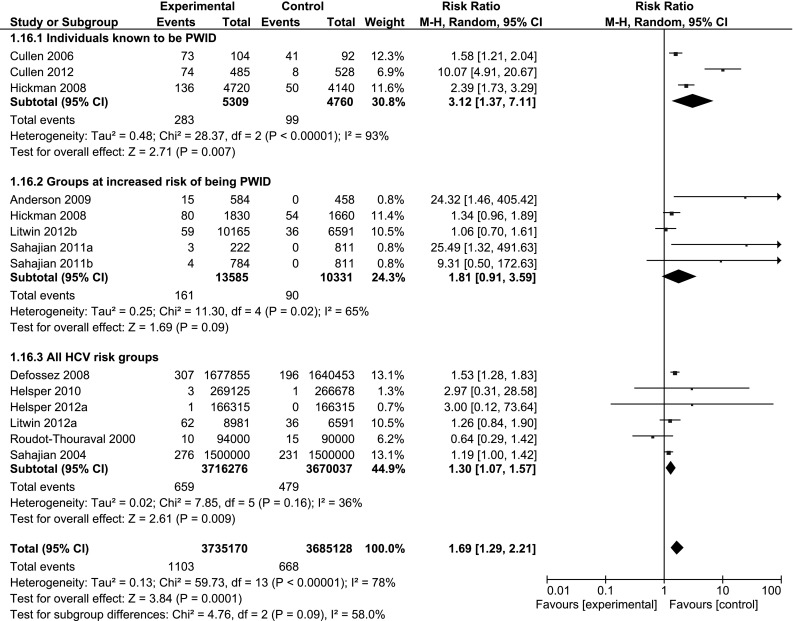
Forest plots comparing targeted HCV testing interventions versus no targeted testing intervention by target group: outcome; HCV antibody cases detected

Targeting of individuals known to be PWID, compared to no targeted testing, increased the number of tests, and the number who tested positive for HCV (n = 4; RR 3.4, 95 % CI 1.7, 6.8; I^2^ = 91 %, and n = 3;3.1, 95 % CI 1.4, 7.1; I^2^ = 93 % respectively). Targeting of specific groups at increased risk of being PWID increased the number of tests (n = 6; RR 5.6, 95 % CI 2.8, 11.4; I^2^ = 97 %), more than the number of positive tests (n = 5; RR 1.8, 95 % CI 0.9, 3.6; I^2^ = 65 %). Targeted testing of individuals with any risk factor for HCV, compared to no targeted testing, was less effective in both increasing the number of tests (n = 6; RR 1.6, 95 % CI 0.9, 2.8; I^2^ = 100 %) and the number of positive tests (n = 6; 1.3, 95 % CI 1.1, 1.6; I^2^ = 36 %) (Fig. 4). Due to the small number of studies that reported on the treatment and care outcomes, stratified analyses were conducted for testing outcomes only.

### Anticipated absolute effects of targeted testing interventions

Targeted HCV testing interventions, compared to no targeted testing, are anticipated to increase the number of people tested for HCV antibody by 112 (95 % CI 59–186) per 10,000 eligible population, and the number of HCV antibody positive cases detected by 1 (95 % CI 0–2) case per 10,000 eligible population. Among the HCV positive population, testing interventions are anticipated to increase the number of people attending specialist appointments by 3,683 (95 % CI 1,274–8,294) per 10,000, and to increase the number commencing HCV treatment by 197 (95 % CI 53–785) per 10,000 population.

## Discussion

This systematic review and meta-analysis provides for the first time a quantitative assessment of the effectiveness of targeted HCV testing interventions in increasing the uptake of HCV testing, treatment, and care. Our review examined more outcomes and identified more primary studies than previous reviews of testing interventions [16, 35], and also included non-English language and economic evaluation studies. Targeted testing interventions—comprising both practitioner-based and media/information-based strategies—were associated with increased test uptake (pooled RR 2.9, 95 % CI 2.0, 4.2), although the association with HCV case detection was less marked (pooled RR 1.7, 95 % CI 1.3, 2.2). This is to be expected even in the most effective of testing interventions, where testing on the basis of risk (rather than on the basis of symptoms) will increase the proportion of negative tests conducted. Targeted testing interventions were also associated with increased HCV treatment uptake (pooled RR 3.3, 95 % CI 1.1, 10.0), but there was insufficient evidence for improvements in SVR or liver-related morbidity. The latter may be due to the short periods of follow-up used by the primary studies included in the review, and their focus on immediate outcomes of test uptake and case detection. While further studies examining the longer-term impact of testing would be desirable, such studies are impractical and other data showing treatment leads to SVR, reduced morbidity, and improved survival are already very strong [36, 37].

The success of targeted HCV testing interventions was dependent on both the risk-group targeted, and the type of strategy adopted. Targeting of individuals known to be PWID was associated with increased test uptake and case detection, whereas targeting individuals with any HCV risk factor was less effective. This may be due to the difference in estimated HCV prevalence between studies targeting individual PWID (range 16.8–70.2 %) and studies targeting individuals with any HCV risk factor (range 0.4–3.0 %).

Studies targeting groups at increased risk of PWID (e.g. homeless persons, or selected birth cohorts) improved test uptake, but there was less evidence for an increase in case detection. This could be because within a group-targeting strategy, those individuals at lower risk are more likely to agree to testing, whereas those at higher risk may not respond to the offer of a test unless they are questioned about their history of risk behaviour.

Practitioner-based studies were effective in increasing test uptake and cases detected, but media/information-based studies were less effective. There was limited detail of the types of interventions employed in the media/information-based studies, but it may be that these interventions were not sufficiently intensive, or only raised awareness of HCV, rather than providing practical information on testing programmes. Information campaigns rely on individuals to self-assess their risk, and former PWID in particular may not self-identify as being part of a risk group, particularly if their exposure was not recent. It has also been suggested that the impact of media campaigns may be short-lived [38], and therefore any positive effects may have been missed in studies that evaluated the campaign some months or years later [21, 22, 33].

There was considerable heterogeneity across the two testing outcomes (test uptake and HCV cases detected), which could not be accounted for by the variables examined in the stratified analyses. A review of the forest plots for these outcomes demonstrated that this heterogeneity derived from variable precision and different effect sizes pointing in the same direction, rather than from directly contradictory findings. The variable precision observed here is due to the vastly different sizes of population denominator used by the different studies (from small clinic-based studies to population-level interventions), leading to very narrow confidence around some estimates, and thus minimal cross-over with other studies. The range in positive effect sizes is likely to be due to the variability in targeting strategies used, as well as the heterogeneity of the intervention and comparison groups across the studies. For example, in the two studies that targeted PWID through GP practices, Cullen et al. [25] identified their target group by asking GPs to recruit current methadone users, whereas Cullen et al. [29] identified PWID through medical records that suggested a history of injecting. The testing intervention in Cullen et al. [25] appeared considerably less effective, because baseline HCV testing among methadone users in GP settings was already very high. Similarly, of two studies that targeted a birth cohort living in an area of socioeconomic deprivation, Anderson et al. was more successful in detecting HCV cases than Litwin et al., possibly due to less routine testing and higher HCV prevalence in the Anderson et al. study.

Across the four studies that reported on treatment and care outcomes [25, 28, 29, 31], 64 % of the estimated chronic HCV population attended a specialist appointment, but only 11 % commenced treatment within a median of 2 years of follow-up. While allowing for the short follow-up period, this suggests that uptake of treatment (in the context of interferon based therapies) within testing interventions is likely to be low, and considerably lower than assumptions used in various studies modelling the cost-effectiveness of testing [22, 39, 40]. In comparison, attendance at specialist appointments was relatively high, suggesting that patients who attended appointments were assessed as unsuitable for treatment, due to patient preference, provider preference, or co-morbidities such as mental health or substance use. It is important that testing interventions provide adequate pre-test counselling, to allow patients to understand the implications of a positive test and the treatments available. In addition, treatment services need to be ready to manage ‘screened’ populations, who may be inherently different to patients who have presented spontaneously for testing.

The majority of the targeted interventions reported in this review were conducted in General Practice settings, of which most were conducted in countries (UK, France, and Ireland) where primary healthcare provision is universal. Current or former PWID are likely to have better access to universal health systems, and this may have contributed to the success of testing interventions in these settings. It should also be noted that all of the primary studies included in this review were based in high-income countries, and the applicability of these results to LMIC is therefore uncertain. Although there is a lack of evidence for targeted HCV testing interventions in these settings, a recent review of HIV testing interventions in LMIC concluded that provider-initiated HIV testing could be effective in increasing test uptake, although the impact on treatment uptake and risk behaviour was equivocal [41]. The HIV-testing studies were based in a number of countries across sub-Saharan Africa and Asia, and delivered testing through hospital outpatient clinics, methadone programmes, and sexual health services. It is therefore probable that HCV testing interventions would be similarly feasible in a range of different LMIC settings. In addition, it might be expected that the relative effect of testing interventions would be even greater in LMIC settings than reported here, given that baseline testing and treatment is likely to be very low.

It is anticipated that approaches to HCV case-finding will undergo considerable changes in the future, as a result of advances in HCV testing, treatment, and care. These include the introduction of rapid testing (providing access to on-the-spot testing and diagnosis for hard-to-reach populations), and the advent of new interferon-free therapies, which will have increased tolerability and efficacy compared to previous regimens. As knowledge and awareness of these developments increase, it is likely that there will be increased willingness, from both providers and patients, to test for HCV and to seek assessment for treatment. This review captures the effectiveness of testing interventions during the era of interferon-based therapies, and the effect sizes quoted here are therefore likely to under-estimate the future effectiveness of testing interventions in the interferon-free era.

This meta-analysis provides for the first time a quantitative assessment of targeted HCV testing interventions, demonstrating that these strategies were effective in diagnosing cases and increasing treatment uptake. Strategies involving practitioner-based interventions yielded the most favourable outcomes. While evidence is lacking on longer-term outcomes, data from studies of treated patients provides strong evidence that increased treatment uptake would translate into improved SVRs, and subsequently to reductions in liver-related morbidity. It is therefore recommended that testing should be targeted at and offered to individuals who are part of a population with high HCV prevalence, or who have a history of HCV risk behaviour.

## Electronic supplementary material

Below is the link to the electronic supplementary material.

Supplementary material 1 (DOCX 23 kb)
