# Supplementary material for: Targeted hepatitis C antibody testing interventions: a systematic review and meta-analysis
Source: Eur J Epidemiol. 2014 Nov 11;30(2):115–29. doi: 10.1007/s10654-014-9958-4 (PMC4366568; doi:10.1007/s10654-014-9958-4)
Supplement: Supplementary file 1 — Supplementary material 1 (DOCX 23 kb) [file 10654_2014_9958_MOESM1_ESM.docx]

### Appendix 1: Search syntax in Medline and Embase

1. *Hepatitis C/

2. HCV.ti,ab.

3. hepatitisc.ti,ab.

4. 1 or 2 or 3

5. *Mass Screening/

6. casefinding.ti,ab.

7. caseidentification.ti,ab.

8. casedetection.ti,ab.

9. screening.ti,ab.

10. test* for hepatitis C.ti,ab.

11. test* for HCV.ti,ab.

12. hepatitis C testing.ti,ab.

13. HCV testing.ti,ab.

14. 5 or 6 or 7 or 8 or 9 or 10 or 11 or 12 or 13

15. 4 and 14

16. limit 15 to humans

17. limit 16 to yr="1994 -Current"

18. remove duplicates from 17

Appendix 2: Sensitivity analyses

| **Outcome** | **Sensitivity analysis on:** | **Inclusion criteria** | **Pooled effect size** | **I ^2^** |
| --- | --- | --- | --- | --- |
| Tested for HCV | Type of study design | **RCT and non-RCT** | **2.90 (2.01, 4.17)** | **100%** |
|  |  | RCT | 2.70 (1.44, 5.05) | 97% |
|  | Inclusion of Defossez 2008 study | **Included** | **2.90 (2.01, 4.17)** | **100%** |
|  |  | Excluded | 2.65 (1.99, 3.52) | 98% |
|  | Estimating the population denominator in Roudot-Thoraval 2000 study | **Best estimate** | **2.90 (2.01, 4.17)** | **100%** |
|  |  | Lowest likely denominator | 2.90 (2.01, 4.17) | 100% |
|  |  | Highest likely denominator | 2.90 (2.02, 4.17) | 100% |
| HCV positive cases detected | Type of study design | **RCT and non-RCT** | **1.66 (1.27, 2.16)** | **76%** |
|  |  | RCT | 1.55 (1.09, 2.19) | 52% |
|  | Inclusion of Defossez 2008 study | **Included** | **1.66 (1.27, 2.16)** | **76%** |
|  |  | Excluded | 1.77 (1.24, 2.43) | 77% |
|  | Estimating the population denominator in Roudot-Thoraval 2000 study | **Best estimate** | **1.66 (1.27, 2.16)** | **76%** |
|  |  | Lowest likely denominator | 1.66 (2.17, 2.16) | 76% |
|  |  | Highest likely denominator | 1.66 (1.27, 2.16) | 75% |
| Referral to HCV specialist | Type of study design | RCT and non-RCT | 6.50 (2.23, 18.97) | 55% |
|  |  | **RCT** | **3.01 (1.79, 5.07)** | **1 study** |
|  | Estimating HCV prevalence for the studies (Cullen 2006) included in this outcome | **Best estimate of prevalence** | **3.01 (1.79, 5.07)** | **1 study** |
|  |  | Highest likely prevalence | 3.02 (1.77, 5.14) | 1 study |
|  |  | Lowest possible prevalence | 3.01 (1.79, 5.07) | 1 study |
| Attendance at HCV specialist | Type of study design | RCT and non-RCT | 6.00 (2.67, 13.48) | 21% |
|  |  | **RCT** | **3.66 (1.92, 6.99)** | **1 study** |
|  | Estimating HCV prevalence for the studies (Cullen 2006) included in this outcome | **Best estimate of prevalence** | **3.66 (1.92, 6.99)** | **1 study** |
|  |  | Highest likely prevalence | 3.67 (1.90, 7.07) | 1 study |
|  |  | Lowest possible prevalence | 3.66 (1.92, 6.99) | 1 study |
| HCV treatment commenced | Type of study design | **RCT and non-RCT** | **3.25 (1.06, 9.95)** | **0%** |
|  |  | RCT | 4.45 (0.53, 37.12) | 1 study |
|  | Estimating HCV prevalence for the studies (Anderson 2009, Cullen 200, Cullen 2012, Lewis 2012) included in this outcome | **Best estimate of prevalence** | **3.25 (1.06, 9.95)** | **0%** |
|  |  | Highest likely prevalence | 3.23 (1.05, 9.93) | 0% |
|  |  | Lowest possible prevalence | 2.90 (0.96, 8.71) | 0% |
| SVR | Type of study design | **RCT and non-RCT** | **1.35 (0.26, 7.09)** | **0%** |
|  |  | RCT | No studies | - |
|  | Estimating HCV prevalence for the studies (Anderson 2009, Cullen 2012) included in this outcome | **Best estimate of prevalence** | **1.35 (0.26, 7.09)** | **0%** |
|  |  | Highest likely prevalence | 1.35 (0.25, 7.12) | 0% |
|  |  | Lowest possible prevalence | 1.37 (0.26, 7.08) | 0% |
